# Transferrin-Conjugated Nanostructured Lipid Carriers for Targeting Artemisone to Melanoma Cells

**DOI:** 10.3390/ijms25169119

**Published:** 2024-08-22

**Authors:** Njoud Altuwaijri, Eman Atef

**Affiliations:** 1 Pharmaceutical Sciences Department, MCPHS University, 179 Longwood Ave, Boston, MA 02115, USA; 2 Pharmacy College, West Coast University, 590 N Vermont Ave, Los Angeles, CA 90005, USA

**Keywords:** lipid nanoparticles (LNPs), transferrin, targeted drug delivery, repurposing, nanostructured lipid carriers (NLCs), cancer

## Abstract

We report a successful formulation of Artemisone (ATM) in transferrin (Tf)-conjugated nanostructured lipid carriers (NLCs), achieving nearly a five-times increase in cell toxicity. The escalating cost of new drug discoveries led to the repurposing of approved drugs for new indications. This study incorporated Artemisone, an antimalarial drug, into a nanostructured lipid carrier (NLC) and tested for possible anticancer effects. The aim was to develop NLCs, and transferrin-conjugated NLCs (NLC-Tf) encapsulating Artemisone to enhance its delivery and anticancer activity. NLC formulations were prepared using high-pressure homogenization followed by ultrasonication and were characterized by particle size, zeta potential, and PDI. The conjugation of (Tf) to (NLC) was confirmed using IR, and the anticancer activity was tested using MTS assay. All formulations were in the nanometer size range (140–167 nm) with different zeta potential values. IR spectroscopy confirmed the successful conjugation of transferrin to NLC. Upon testing the formulations on melanoma cell lines using MTS assay, there was a significant decrease in viability and an increase in the encapsulated ATM-Tf toxicity compared to positive control ATM. The NLCs presented a promising potential carrier for delivering ATM to melanoma cells, and further conjugation with Tf significantly improved the ATM cytotoxicity.

## 1. Introduction

With the increased cost of new drug investigations, some pharmaceutical companies are considering drug repurposing or repositioning. This involves investigating new uses and applications for already approved drugs, thereby shortening development timeframes and reducing total development costs. This was evident in the early stages of COVID-19 when numerous institutions were rigorously investigating the efficacy of already approved drugs against the virus [1,2,3].

Artemisinins, a class of antimalarial drugs, exhibit potential for repurposing as anticancer medications. The groups’ major challenge of use is poor solubility and permeability (BSC IV). Scientists developed three generations of artemisinins to overcome these challenges, including semisynthetic and fully synthetic compounds [4].

Several studies have investigated the mechanism of action of Artemisone (ATM) as a promising anticancer drug for use in various combinations [5,6]. Our focus is to improve the delivery and, thus, the efficacy of Artemisone by incorporating it in a nanostructured lipid carrier conjugated to a targeting ligand. One of the suggested anticancer mechanisms of action of artemisinin is the reaction between the iron in cancer cells and the endoperoxide moiety of artemisinins [5,7,8,9,10], cleavage of the endoperoxide bridge releases ROS, leading to a series of processes that kill cancer cells [11].

Artemisone aqueous solubility at pH 7.4 is 89 μg/mL, and its log P at pH 7.4 is 2.49. The low lipophilicity of ATM has contributed to its low cytotoxicity and neurotoxicity in different in vivo and in vitro assays [4,12]. The organic solvent-free first-generation solid lipid nanoparticles (SLNs) were a nanoparticle breakthrough, yet their high crystallinity resulted in a significant drug-loading challenge. The latter challenge was addressed by incorporating liquid lipids in nanostructured lipid carriers (NLCs), resulting in solid matrix imperfections; in other words, a less organized lipid–solid matrix with a higher capacity to incorporate significant amounts of the drug and increased physical stability during storage [13,14,15,16,17]. In addition to these benefits, the lipids and surfactants used in NLCs are FDA-approved. Another benefit is that the NLC preparation is simple and easily scaled up [18,19].

Nanoparticles are frequently used to facilitate the delivery of hydrophobic drugs, improve their pharmacokinetic properties, and subsequently enhance their effect [20,21]. In addition, a major formulation advantage of nanoparticles is their ability to target the anticancer drug to the tumor by the addition of targeting ligands such as transferrin. These nanocarrier features eventually change the biodistribution of the anticancer agent, favoring its accumulation at the tumor site [22,23,24].

Transferrin receptors are surface homodimers responsible for iron uptake and cell growth regulation. Fe (III) is solubilized by binding to Tf. It forms holo-transferrin (holo-Tf), and then is transported into the cells by TfR [25]. The binding of the holo-Tf to the TfR forms an internalized complex into the cell through endocytosis. Next, the iron is released from Tf due to the low pH, the Tf–TfR complex is recycled back to the surface, and, finally, Tf is released [26,27].

The codelivery of Tf and artemisinins in one system results in the internalization of Tf, iron, and artemisinins into the cell by endosomes in one entity. Once iron is released inside the cell, artemisinin activation starts, producing ROS, resulting in mitochondrial damage, caspase activation, and cell death [28,29,30].

Scientists have conducted studies using Tf as a targeting ligand with artemisinins and approved their higher efficacy over nontargeted formulations against different cancers [30,31]. In summary, transferrin emerges as a promising targeting ligand when conjugated with NLCs for encapsulating Artemisone against melanoma, aligning with the mechanism of action typical of artemisinins. The novelty of our research lies in utilizing the cancer cells’ iron uptake mechanism by transferrin receptors and the endoperoxide moiety of the drug to enhance its uptake and toxicity. Utilizing Tf-conjugated NLCs shows potential as an effective carrier for delivering Artemisone to melanoma cells, enhancing cytotoxicity through synergistic features. This study aimed to develop NLCs and transferrin-conjugated NLCs encapsulating Artemisone to facilitate its delivery and improve its anticancer activity against melanoma.

## 2. Results

### 2.1. Preparation and Characterization of NLC Formulation

The characterization of different tested NLC formulations is presented in Table 1 and Table 2. Formulation A yielded the smallest particle size with an acceptable polydispersity index and zeta potential. As a result, it was chosen to be used throughout the study. The parameters necessary to evaluate NLC formulations are particle size, zeta potential, polydispersity index, and encapsulation efficiency, and they are presented in Table 3.

### 2.2. Coupling Efficiency

No variations in coupling efficiency were observed upon varying the ratios of lipid to Tf (95:5, 90:10, 80:20). The ratio of lipid to Tf used in the study was 90:10, which was reported in several studies [32,33]. The percent of Tf coupled to NLC was 30% for NLC-TF and 26% for NLC-TF-ATM. No significant difference was found between the percent of Tf coupled to NLC-TF and NLC-TF-ATM. The statistical differences were tested using a *t*-test.

### 2.3. IR Spectroscopic Analysis

**Figure 1:**
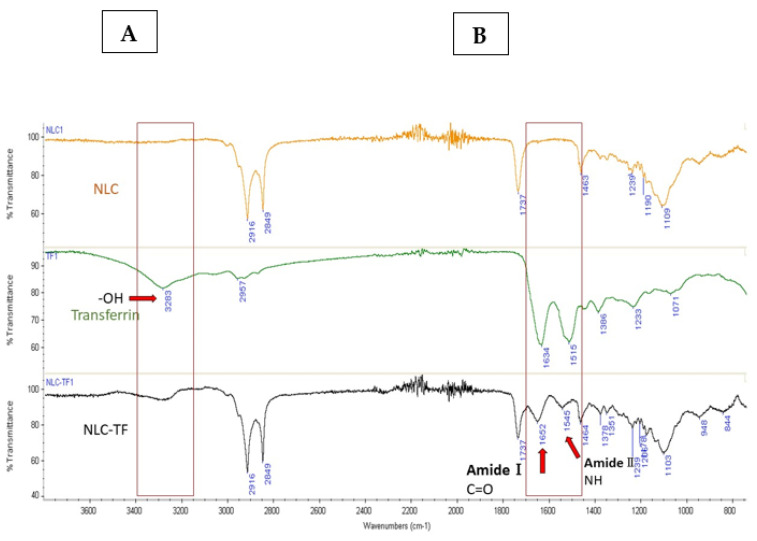
The IR spectra of NLC, TF, and NLC-TF. Panels (**A**,**B**) define the peak range where changes due to conjugation are observed. The amide bond formation in the NLC-TF spectrum confirmed the conjugation of Tf to the NLC. The presence of amide Ⅰ bond at 1652 cm^−1^ (C=O stretching) and amide Ⅱ at 1545 cm^−1^ (CN stretching and NH bending), as well as the reduction in the carboxylic peak at 3283 cm^−1^, confirms a conjugation reaction (n = 3).

The IR spectra of the formulations NLC, NLC-TF, and transferrin are presented in Figure 1 and the IR identification key peaks are present in Table 4.

### 2.4. In Vitro Cytotoxicity Study

**Figure 2:**
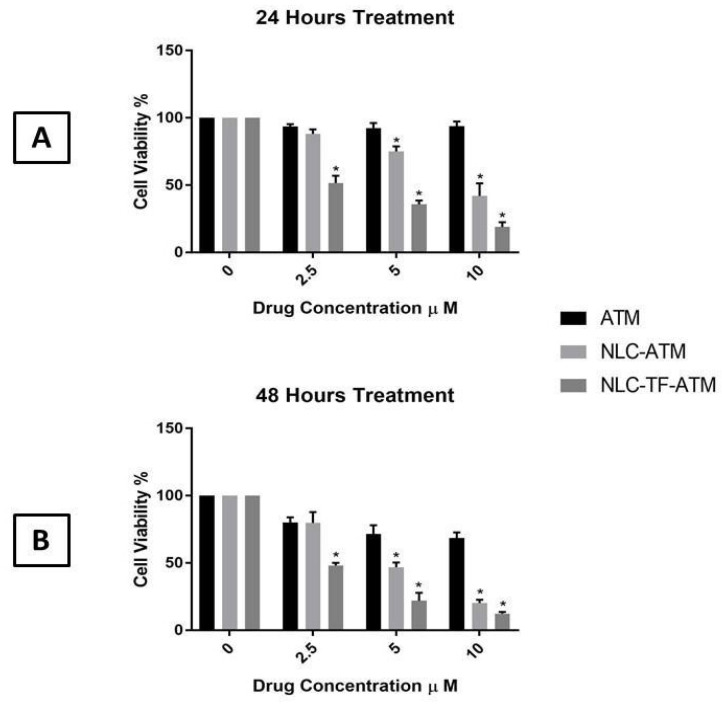
Cell viability of SK-MEL-19 cells after treatment with Artemisone (ATM), NLC-ATM, and NLC-TF-ATM (**A**) after 24 h of treatments; (**B**) after 48 h of treatment. Data are plotted as mean ± SD (n = 3) (*) *p* < 0.001.

The MTS assay results of ATM, NLC-ATM, and NLC-TF-ATM on SK-MEL-19 after 24 and 48 h are shown in Figure 2.

The results indicate that the cell viability of SK-MEL-19 decreased significantly after treatment with NLC-ATM and NLC-TF-ATM compared to free ATM using 5 and 10 µM after 24 and 48 h. For the 2.5 µM concentration, the cell viability after treatment with NLC-TF-ATM was significantly reduced compared to NLC-ATM and ATM treatment after 24 and 48 h.

An increase in toxicity by almost five times was observed with the drug upon encapsulation and conjugation with Tf compared to the drug alone at both 24 h and 48 h. The significance of the results was determined using one-way ANOVA followed by Tukey’s test.

### 2.5. Stability Studies

The NLC and NLC-ATM formulations maintained their stability under both 4 °C and room temperature for 30 days, while the NLC-TF and NLC-TF-ATM formulations were only stable when stored at 4 °C. There was a significant increase in the particle size of the NLC-TF and NLC-TF-ATM formulations stored at room temperature after seven days. A reduction in the zeta potential and an increase in the polydispersity index was also observed. Storing the formulations at 4 °C proved to be suitable for maintaining the stability of all formulations.

## 3. Discussion

We have demonstrated that ATM encapsulation into the NLCs increased cell internalization and improved its anticancer effect by increasing its accumulation and cellular uptake using Tf-conjugated NLCs. ATM has two challenging properties: low solubility and short half-life [12]. The NLCs were used to encapsulate the drug and deliver it to melanoma cells to improve ATM’s efficacy and physicochemical properties.

In addition, the Tf conjugation increased cell uptake and, hence, drug toxicity, which could be due to the uptake mechanism of the NLC-TF by TfR on cancer. The abundance of the TfR receptors on the cancer cell’s surface and the high iron uptake contributed to the selective cytotoxicity of artemisinins toward cancer cells and not normal cells [28], in addition to other mechanisms that have not yet been investigated.

The homogenization followed by ultrasonication used in this study is the most commonly used preparation method for NLCs [34]. It yields a small particle size formulation with a narrow distribution and is prepared in aqueous media. The particle size of the NLC and NLC-TF formulations in this study was in the nanometer size range below 200 nm, which is recommended in anticancer treatments to utilize the EPR effect [35,36]. After conjugating the NLCs with Tf, the particle’s size did not change significantly.

The zeta potential of the NLCs and NLC-ATMs was around +15 mV; however, after conjugation with Tf, it changed to −19 mV. These zeta potential values are expected to result in moderate stability because for the formulations to be stable, a zeta potential > ±30 mV is recommended by some references [37,38].

Our studies confirmed the stability of the formulations for up to 30 days at 4 °C even with low zeta potential. When considering the stability of nanoparticle formulations, the particle size, in addition to the zeta potential, plays an important role [39]. Several published studies have demonstrated stable formulations of nanostructured lipid carriers (NLCs) despite having a low zeta potential. These findings underline the complexity of factors influencing the stability of NLCs [32,40,41,42].

Highly cationic nanoparticles, with positive potential, are well known to interact strongly with cell membranes, resulting in higher uptake but more toxicity [39]. Our NLC formulations had positive zeta potential, while the NLC-TF possessed a negative charge. The effect of the charge on the interaction with cell membranes and toxicity would need further in vivo investigation.

Another critical parameter for characterizing NLCs is the polydispersity index (PDI), analyzed by dynamic light scattering technique, which indicates the particles’ homogeneity and monodispersity. In our study, the PDI values of different formulations were around 0.2 ± 0.1, a value that is commonly acceptable for lipid-based nanocarriers. A PDI below 0.25 is generally regarded as evidence of monodispersity and homogeneity in lipid nanocarrier formulations, as reported by several references [43,44,45]. Particle size, PDI, and zeta potential are crucial parameters that collectively influence the stability, size distribution, and surface charge of the nanostructure. Controlling these parameters plays an important role in the pharmacokinetics of the nanocarriers and, eventually, their clinical applications [43,46].

A relatively high drug loading of 90%, a known advantage of NLCs, was achieved before conjugation. However, upon conjugation, the encapsulation efficiency dropped to 60%. Similar phenomena were reported by Khajavinia et al. upon conjugating Tf to the NLC-encapsulated etoposide [47]. According to several studies, the drug leaking during the conjugation process may be the cause of the decrease in drug loading following conjugation [32,47].

**Figure 3:**
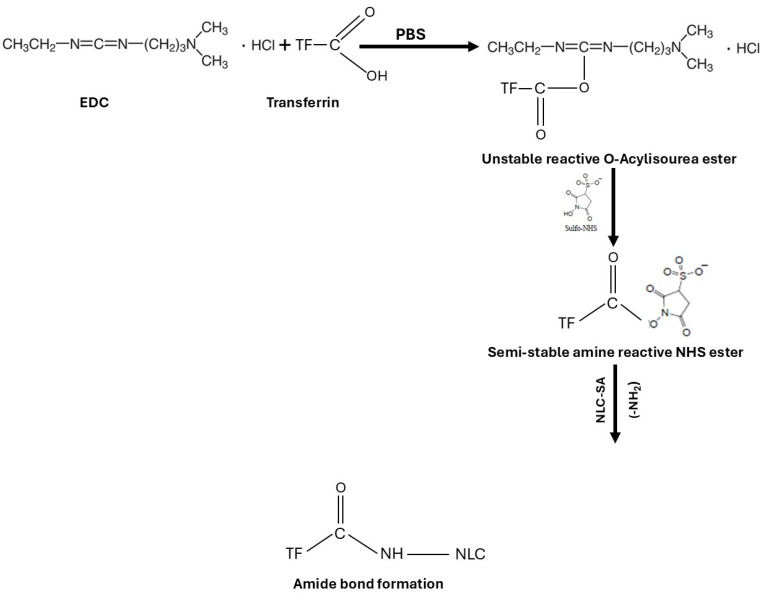
Schematic representation of transferrin conjugation with NLCs using EDC/NHS coupling reaction.

The FT-IR is used to confirm the chemical reaction between the amine of the SA in NLCs and the carboxylic acid in Tf to form an amide bond. The coupling reaction was conducted using EDC as a coupling agent and NHS as an additive to stabilize the reaction in PBS. EDC activates the carboxyl group by forming an amine-reactive O-acylisourea intermediate. The intermediate is unstable in an aqueous solution and needs to be reacted quickly with the amino group to form an amide bond and release iso-urea byproducts. If the intermediate fails to react with the amine, a hydrolysis reaction will occur, and the carboxyl group will be regenerated. NHS is added to the coupling reaction to stabilize the intermediate and allow the reaction between the carboxylic acid and the amino group (Figure 3). For the amide reaction to succeed, the amination reaction should be faster than the hydrolysis of the activated carboxylic acid intermediate [48,49,50].

The spectra of NLC-TF in Figure 1 show the two amide peaks at 1652 and 1545 cm^−1^, which confirm the conjugation of Tf to the SA in the NLC formulations. In reference to peaks 2916 and 2849 cm^−1^, it is apparent that most NLC peaks represent the peak composition of NLC-TF. As a result, the 1652 and 1545 cm^−1^ are new peaks, which are expected due to the small weight percentage of TF compared to NLC (Table 4). The presence of amide peaks on different nanoparticle formulations was reported in different studies after conjugation with Tf [47,51].

In the cell viability assay experiments, it was observed that encapsulating the ATM into NLC and NLC-Tf significantly increased its cytotoxic effect at lower concentrations. Using anticancer drugs at lower concentrations would reduce some side effects as well as reduce the toxicity on normal cells. We conclude from these experiments that encapsulating the drug into a nanoparticle would increase its internalization into the cells, which could be due to its size and lipid nature. In addition, conjugating the NLCs with Tf caused higher toxicity to the cells, and that could be due to the uptake mechanism of the NLC-TFs by TfR on cancer cells. The hypothesis suggested by Sahoo and Labhasetwar could explain the increase in the cytotoxicity of conjugated NLCs [52]. They stated that the TF-conjugated nanoparticles could have different intracellular sorting pathways after their uptake by TfR, compared to the unconjugated nanoparticles. That specific pathway would increase the accumulation of the conjugated nanoparticles inside the cells and increase therapeutic efficacy. Different studies confirmed the increase in the cytotoxicity after conjugating their nanoparticle formulations with Tf. Leto et al. demonstrated an increase in the cytotoxicity of artemisinin encapsulated into a liposome coupled to Tf by the terminal PEG. There was a reduction in the IC50 from 127 ± 8.5 μM for the free drug to 69 ± 23 μM for the TF-conjugated liposome [30]. In conclusion, different studies on different nanoparticle formulations conjugated with Tf have demonstrated an increase in the anticancer activity of the drugs encapsulated [40,49,50,51,52].

## 4. Materials and Methods

### 4.1. Preparation of Nanostructured Lipid Carriers (NLCs) Formulations

The nanostructured lipid carriers were prepared by homogenization followed by ultrasonication. Different ratios of solid lipids, liquid lipids, and surfactants were evaluated before finalizing the optimum formulation. The lipid phase composed of Compritol^®^, Labrafil^®^ (Gattefosse, Paramus, NJ, USA), and lecithin (Letco Medical, Decatur, AL, USA), with or without ATM (Alsachim, Graffenstaden, France), was heated to melt at 80 °C; simultaneously, the aqueous phase containing Tween-80 (Letco Medical, Decatur, AL, USA) was heated to the same temperature. Next, the aqueous phase was dispersed in the lipid phase and mixed using a homogenizer (Ultra-turra T8 homogenizer, Ika, Germany) at 3000 RPM for 3 min. The resulting pre-emulsion was ultrasonicated under ice using a Qsonica ultrasonic processor Q700 (Qsonica, Newtown, CT, USA) with a microtip at an amplitude of 80 for 10 min. The sonication was at a pulsation mode of 20 s ON followed by 10 s OFF, repeated for 10 min. The resulting nanoemulsion was cooled in an ice bath for flash crystallization, leading to the formation of the nanoparticles. The optimum formulation was chosen according to the acceptable particle size, zeta potential, and PDI.

### 4.2. Transferrin NLC Conjugation

In order to conjugate Tf (Sigma-Aldrich St. Louis, MO, USA) with NLC formulations, stearyl amine (SA) (Sigma-Aldrich St. Louis, MO, USA) was added to the above-prepared lipid phase. The primary terminal amine of SA conjugates with Tf and forms an amide bond. The SA was added in the lipid phase with Compritol^®^, Labrafil^®^, and lecithin, followed by the same preparation method. Transferrin was conjugated to the NLC through a modified coupling reaction reported by Gupta et al. [32]. The carboxyl group of Tf was covalently coupled with SA in the NLC formulations in a ratio of 90:10 w/w (lipids: Tf). EDC (TCI America, Portland, OR, USA) was added as a coupling agent, as well as NHS (Sigma-Aldrich St. Louis, MO, USA), which improves the efficiency of the coupling reaction. First, Tf, EDC, and NHS were dissolved in PBS using a magnetic stirrer for 15 min. Next, either NLC-SA or NLC-ATM-SA was added to the mixture and stirred for 24 h. Ultrafiltration (0.22 µm) was used to remove the excess TF, EDC, and NHS. Dialysis of the formulation in distilled water for 1 or 2 h using a dialysis bag was investigated as a method to remove excess Tf, EDC, and NHS. This method was excluded as 15% of the drug was released after 2 h of dialysis.

### 4.3. Coupling Efficiency

The coupling of Tf to NLC-SA was quantified using Bradford protein assay (Bio-Rad, Hercules, CA, USA). Using ultrafiltration, the unconjugated Tf was separated and analyzed. The average quantity of Tf coupled to the NLC was calculated indirectly by subtracting the unconjugated Tf in the filtrate from the initial amount of Tf added.

### 4.4. Characterization of Nanostructured Lipid Carrier (NLC) Formulations

#### 4.4.1. Particle Size, Zeta Potential, and Polydispersity

The mean particle size, zeta potential, and polydispersity index of the NLC formulations were determined by dynamic light scattering using a Brookhaven 90 plus size analyzer (Brookhaven instruments, Holtsville, NY, USA). (n = 3)

#### 4.4.2. Encapsulation Efficiency

The encapsulation efficiency was calculated by determining the free drug in the medium using ultrafiltration. Samples from NLC-ATM and NLC-TF-ATM of 500 µL were transferred to a centrifugal filter with a pore size of 0.1 µm for centrifugation. The samples were centrifuged at 1844 g for 12 min to separate the free ATM using 0.1 µm ultrafilters and then analyzed by HPLC. The entrapment efficiency was calculated from the following equation:

(1) $$Encapsulation\;efficiencyEE(\%)=\frac{\left(W\;initial\;drug-W\;unbound\;drug\right)}{W\;initial\;drug}\times 100$$

### 4.5. FT-IR Analysis

FT-IR confirmed the conjugation of Tf with SA in the NLC formulations.

The spectra of the lyophilized samples of NLC, NLC-ATM, NLC-TF, NLC-TF-ATM, Compritol^®^, SA, and Tf were collected using Nicolet FT-IR spectrometer iS series from Thermo Scientific (Thermo Scientific, Waltham, MA, USA).

### 4.6. In Vitro Cytotoxicity of NLC-TF-ATM and Its Components

The cytotoxicity of ATM in DMSO, NLC-ATM, NLC-TF-ATM, Labrafil^®^, tween80, and lecithin was assessed against skin melanoma cell line (SK-MEL-19) (Sloan Kettering Institute, New York, NY, USA) using MTS assay (Promega, Madison, WI, USA). Two other cell lines, A375 and PC3, were also investigated at the beginning of the study. The cells were seeded at 5 × 10^3^ cells/well in 96-well culture plates for 24 h. Next, the cells were treated with the different NLC formulations as well as the NLC components at different concentrations. The plates were incubated for 24 and 48 h, and then 20 µL of the MTS reagent was added. The plates were incubated for four hours, and the absorbance was measured at 490 nm by a microplate reader (Synergy HT, BioTek, Winooski, VT, USA), where the untreated cells were used as a control with 100% cell viability. The following equation was used to calculate cell viability:

(2) $$Cell\;viability\%=\frac{Mean\;absorbance\;of\;the\;sample}{absorbance\;of\;the\;control}\times 100$$

### 4.7. Lyophilization

Lyophilization of different NLC samples was performed using a Labconco FreeZone triad cascade benchtop freeze-dryer (Labconco, Kansas City, MO, USA). The samples were prefrozen at −80 °C for 24 h and then freeze-dried at −25 °C for 48 h.

### 4.8. Stability Studies

Samples of NLC, NLC-ATM, NLC-TF, and NLC-TF-ATM were stored at different storage conditions, room temperature, and 4 °C. Characterization of the size, PDI, and zeta potential was performed on diluted samples on days 0, 7, 14, and 30 using a particle size analyzer (n = 3).

### 4.9. Statistical Analysis

Statistical analysis was accomplished using analysis of variance (ANOVA) and *t*-test followed by Tukey’s test. Differences are considered statistically significant at a level of *p*-value < 0.01.

## 5. Conclusions

We conclude that transferrin-conjugated nanostructured lipid carriers (NLC-Tfs) are a promising delivery system for Artemisone (ATM), a repurposed antimalaria drug, to melanoma cells. The medication’s cytotoxicity significantly increased with the conjugation of Tf to NLCs, suggesting that this carrier might deliver various chemical compounds to cancer cells. NLCs are an efficient drug delivery system capable of addressing challenging drug properties and have the potential for scalable industrial production. Hence, additional research is necessary to evaluate this delivery system’s safety and effectiveness in animal models. These results highlight the value of repurposing medications and the potential of nanotechnology to enhance medication efficacy and delivery.

## Figures and Tables

**Table 1:** Characterization of NLC formulations with different surfactants (Tween 80 or Kolliphor).

Formulation	Tween 80 (mg)	Kolliphor (mg)	Particle Size (nm)	Polydispersity Index
* A	200	0	140	0.2
B	50	50	315	0.4
C	25	25	420	0.4
D	25	0	500	0.5
E	0	25 or 50	**	
F	0	100	>1 µm	**
G	0	200	>1 µm	**

** Very large particle size or polydispersity index that the particle size analyzer cannot measure. * Optimum formulation used in the study. All formulations contained the same amounts of lipids, surfactant, and water (compritol^®^ 200 mg, labrafil^®^ 200 mg, lecithin 50 mg, water 10 mL).

**Table 2:** Characterization of different NLC formulations prepared using different ratios of solid and liquid lipids.

Formulation	Compritol^®^:Labrafil^®^	Particle Size (nm)	Polydispersity Index	Zeta Potential (mV)
1	70:30	168	0.2	+24.9
2	60:40	160	0.2	+20.6
3	50:50	140	0.2	+17.8
4	40:60	150	0.2	+22.3
5	30:70	155	0.2	+20.7

+ A constant weight of lecithin, tween 80, and water were used in the formulations: (lecithin 50 mg, tween 80 200 mg, water 10 mL).

**Table 3:** Characterization of NLC formulations and encapsulation efficiencies of blank, encapsulated, and conjugated NLCs.

Formulation	Particle Size (nm)	Zeta Potential (mV)	Polydispersity Index (PDI)	Encapsulation Efficiency (EE%)
NLC	140 ± 4	+15 ± 0.4	0.2 ± 0.0	__
NLC-ATM	167 ± 15	+15 ± 2	0.2 ± 0.0	95 ± 5%
NLC-TF	167 ± 10	* −19 ± 7	0.2 ± 0.1	__
NLC-TF-ATM	154 ± 5	−18 ± 2	0.2 ± 0.0	60 ± 9%

* The change in the zeta potential charge could be due to the nanoparticle’s coating with Tf, which changed the zeta potential toward a negative value.

**Table 4:** The identification of the IR key peaks.

Material	Peak Number	Peak Assignment	Monitored Peak Changes to Confirm Conjugation
NLC	2915 cm^−1^ 2849 cm^−1^	C-H stretching vibration	
	1736 cm^−1^	carbonyl band -C=O	
	1463 cm^−1^	C=C stretching vibration	
Transferrin	1634 cm^−1^	Amide Ⅰ bond C=O stretching	
	1515 cm^−1^	Amide Ⅱ CN stretching and NH bending	
	3282 cm^−1^	-OH of the carboxylic acid	
NLC-TF	1652 cm^−1^	Amide Ⅰ bond C=O stretching	Appearance
	1545 cm^−1^	Amide Ⅱ CN stretching and NH bending	Appearance
	3283 cm^−1^	-OH of the carboxylic acid	Reduction

## Data Availability

The data that support the findings of this study are available from the corresponding author upon reasonable request.

## Abbreviations

ATM	Artemisone
EDC	1-(3-Dimethylaminopropyl)-3-ethylcarbodiimide Hydrochloride
EE	Encapsulation efficiency
EPR	Enhanced permeability and retention effect
MTS	3-(4:5-Dimethylthiazol-2-yl)-5-(3-carboxymethoxyphenyl)-2-(4-sulfophenyl)-2H-tetrazolium)
NHS	N-Hydroxy succinimide
NLC	Nanostructured lipid carriers
PDI	Polydispersity index
ROS	Reactive oxygen species
SA	Stearylamine
SLN	Solid lipid nanoparticle
Tf	Transferrin
TfR	Transferrin receptor

## References

[1] Ng Y.L., Salim C.K., Chu J.J.H. (2021). Drug repurposing for COVID-19: Approaches, challenges and promising candidates. Pharmacol. Ther..

[2] Kumar S., Roy V. (2023). Repurposing Drugs: An Empowering Approach to Drug Discovery and Development. Drug Res..

[3] Pushpakom S., Iorio F., Eyers P.A., Escott K.J., Hopper S., Wells A., Doig A., Guilliams T., Latimer J., McNamee C. (2018). Drug repurposing: Progress, challenges and recommendations. Nat. Rev. Drug Discov..

[4] Haynes R.K. (2006). From artemisinin to new artemisinin antimalarials: Biosynthesis, extraction, old and new derivatives, stereochemistry and medicinal chemistry requirements. Curr. Top. Med. Chem..

[5] Zhou X.Y., Suo F.Z., Haslinger K., Quax W.J. (2022). Artemisinin-Type Drugs in Tumor Cell Death: Mechanisms, Combination Treatment with Biologics and Nanoparticle Delivery. Pharmaceutics.

[6] Van Huijsduijnen R.H., Guy R.K., Chibale K., Haynes R.K., Peitz I., Kelter G., Phillips M.A., Vennerstrom J.L., Yuthavong Y., Wells T.N.C. (2013). Anticancer properties of distinct antimalarial drug classes. PLoS ONE.

[7] Beekman A.C., Wierenga P.K., Woerdenbag H.J., Van Uden W., Pras N., Konings A.W.T., El-Feraly F.S., Galal A.M., Wikström H.V. (1998). Artemisinin-derived sesquiterpene lactones as potential antitumour compounds: Cytotoxic action against bone marrow and tumour cells. Planta Medica.

[8] Bhaw-Luximon A., Jhurry D. (2017). Artemisinin and its derivatives in cancer therapy: Status of progress, mechanism of action, and future perspectives. Cancer Chemother. Pharmacol..

[9] Crespo-Ortiz M.P., Wei M.Q. (2012). Antitumor activity of artemisinin and its derivatives: From a well-known antimalarial agent to a potential anticancer drug. J. Biomed. Biotechnol..

[10] Firestone G.L., Sundar S.N. (2009). Anticancer activities of artemisinin and its bioactive derivatives. Expert Rev. Mol. Med..

[11] Golenser J., Hunt N.H., Birman I., Jaffe C.L., Zech J., Mäder K., Gold D. (2023). Applicability of Redirecting Artemisinins for New Targets. Glob. Chall..

[12] Nagelschmitz J., Voith B., Wensing G., Roemer A., Fugmann B., Haynes R.K., Kotecka B.M., Rieckmann K.H., Edstein M.D. (2008). First assessment in humans of the safety, tolerability, pharmacokinetics, and ex vivo pharmacodynamic antimalarial activity of the new artemisinin derivative artemisone. Antimicrob. Agents Chemother..

[13] Müller R.H., Radtke M., Wissing S.A. (2002). Nanostructured lipid matrices for improved microencapsulation of drugs. Int. J. Pharm..

[14] Muchow M., Maincent P., Müller R.H. (2008). Lipid nanoparticles with a solid matrix (SLN^®^, NLC^®^, LDC^®^) for oral drug delivery. Drug Dev. Ind. Pharm..

[15] Shidhaye S.S., Vaidya R., Sutar S., Patwardhan A., Kadam V.J. (2008). Solid lipid nanoparticles and nanostructured lipid carriers—Innovative generations of solid lipid carriers. Curr. Drug Deliv..

[16] Thiruchenthooran V., Espina M., Świtalska M., Bonilla-Vidal L., Wietrzyk J., Garcia M.L., Souto E.B., Sánchez-López E., Gliszczyńska A. (2024). Combination of Indomethacin with Nanostructured Lipid Carriers for Effective Anticancer Therapy. Int. J. Nanomed..

[17] Jeitler R., Glader C., König G., Kaplan J., Tetyczka C., Remmelgas J., Mußbacher M., Fröhlich E., Roblegg E. (2024). On the Structure, Stability, and Cell Uptake of Nanostructured Lipid Carriers for Drug Delivery. Mol. Pharm..

[18] Müller R.H., Shegokar R., Keck C.M. (2011). 20 years of lipid nanoparticles (SLN & NLC): Present state of development & industrial applications. Curr. Drug Discov. Technol..

[19] Das S., Chaudhury A. (2011). Recent advances in lipid nanoparticle formulations with solid matrix for oral drug delivery. AAPS PharmSciTech.

[20] Selvamuthukumar S., Velmurugan R. (2012). Nanostructured Lipid Carriers: A potential drug carrier for cancer chemotherapy. Lipids Health Dis..

[21] Estanqueiro M., Amaral M.H., Conceição J., Lobo J.M.S. (2015). Nanotechnological carriers for cancer chemotherapy: The state of the art. Colloids Surf. B Biointerfaces.

[22] Wicki A., Witzigmann D., Balasubramanian V., Huwyler J. (2015). Nanomedicine in cancer therapy: Challenges, opportunities, and clinical applications. J. Control. Release.

[23] Sun T., Zhang Y.S., Pang B., Hyun D.C., Yang M., Xia Y. (2014). Engineered nanoparticles for drug delivery in cancer therapy. Angew. Chem.-Int. Ed..

[24] Hare J.I., Lammers T., Ashford M.B., Puri S., Storm G., Barry S.T. (2017). Challenges and strategies in anti-cancer nanomedicine development: An industry perspective. Adv. Drug Deliv. Rev..

[25] Nakase I., Gallis B., Takatani-Nakase T., Oh S., Lacoste E., Singh N.P., Goodlett D.R., Tanaka S., Futaki S., Lai H. (2009). Transferrin receptor-dependent cytotoxicity of artemisinin-transferrin conjugates on prostate cancer cells and induction of apoptosis. Cancer Lett..

[26] Daniels T.R., Delgado T., Rodriguez J.A., Helguera G., Penichet M.L. (2006). The transferrin receptor part I: Biology and targeting with cytotoxic antibodies for the treatment of cancer. Clin. Immunol..

[27] Daniels T.R., Delgado T., Helguera G., Penichet M.L. (2006). The transferrin receptor part II: Targeted delivery of therapeutic agents into cancer cells. Clin. Immunol..

[28] Nakase I., Lai H., Singh N.P., Sasaki T. (2008). Anticancer properties of artemisinin derivatives and their targeted delivery by transferrin conjugation. Int. J. Pharm..

[29] Lai H., Sasaki T., Singh N.P., Messay A. (2005). Effects of artemisinin-tagged holotransferrin on cancer cells. Life Sci..

[30] Leto I., Coronnello M., Righeschi C., Bergonzi M.C., Mini E., Bilia A.R. (2016). Enhanced Efficacy of Artemisinin Loaded in Transferrin-Conjugated Liposomes versus Stealth Liposomes against HCT-8 Colon Cancer Cells. ChemMedChem.

[31] Zhang H., Ji Y., Chen Q., Jiao X., Hou L., Zhu X., Zhang Z. (2015). Enhancement of cytotoxicity of artemisinin toward cancer cells by transferrin-mediated carbon nanotubes nanoparticles. J. Drug Target..

[32] Gupta Y., Jain A., Jain S.K. (2007). Transferrin-conjugated solid lipid nanoparticles for enhanced delivery of quinine dihydrochloride to the brain. J. Pharm. Pharmacol..

[33] Jain S.K., Chaurasiya A., Gupta Y., Jain A., Dagur P., Joshi B., Katoch V.M. (2008). Development and characterization of 5-FU bearing ferritin appended solid lipid nanoparticles for tumour targeting. J. Microencapsul..

[34] Gomaa E., Fathi H.A., Eissa N.G., Elsabahy M. (2022). Methods for preparation of nanostructured lipid carriers. Methods.

[35] Fang J., Islam W., Maeda H. (2020). Exploiting the dynamics of the EPR effect and strategies to improve the therapeutic effects of nanomedicines by using EPR effect enhancers. Adv. Drug Deliv. Rev..

[36] Shen X., Pan D., Gong Q., Gu Z., Luo K. (2024). Enhancing drug penetration in solid tumors via nanomedicine: Evaluation models, strategies and perspectives. Bioact. Mater..

[37] Bhattacharjee S. (2016). DLS and zeta potential—What they are and what they are not?. J. Control. Release.

[38] Rodriguez-Ruiz V., Salatti-Dorado J.A., Barzegari A., Nicolas-Boluda A., Houaoui A., Caballo C., Caballero-Casero N., Sicilia D., Venegas J.B., Pauthe E. (2018). Astaxanthin-Loaded Nanostructured Lipid Carriers for Preservation of Antioxidant Activity. Molecules.

[39] Lu G.W., Gao P., Kulkarni V.S. (2010). CHAPTER 3—Emulsions and Microemulsions for Topical and Transdermal Drug Delivery. Handbook of Non-Invasive Drug Delivery Systems.

[40] Emami J., Rezazadeh M., Sadeghi H., Khadivar K. (2017). Development and optimization of transferrin-conjugated nanostructured lipid carriers for brain delivery of paclitaxel using Box–Behnken design. Pharm. Dev. Technol..

[41] Mulik R.S., Mönkkönen J., Juvonen R.O., Mahadik K.R., Paradkar A.R. (2012). Apoptosis-induced anticancer effect of transferrin-conjugated solid lipid nanoparticles of curcumin. Cancer Nanotechnol..

[42] Sahoo S.K., Ma W., Labhasetwar V. (2004). Efficacy of transferrin-conjugated paclitaxel-loaded nanoparticles in a murine model of prostate cancer. Int. J. Cancer.

[43] Khajavinia A., Varshosaz J., Dehkordi A.J. (2012). Targeting etoposide to acute myelogenous leukaemia cells using nanostructured lipid carriers coated with transferrin. Nanotechnology.

[44] Grabarek Z., Gergely J. (1990). Zero-length crosslinking procedure with the use of active esters. Anal. Biochem..

[45] Staros J.V., Wright R.W., Swingle D.M. (1986). Enhancement by N-hydroxysulfosuccinimide of water-soluble carbodiimide-mediated coupling reactions. Anal. Biochem..

[46] Badland M., Crook R., Delayre B., Fussell S.J., Gladwell I., Hawksworth M., Howard R.M., Walton R., Weisenburger G.A. (2017). A comparative study of amide-bond forming reagents in aqueous media—Substrate scope and reagent compatibility. Tetrahedron Lett..

[47] Kowalczyk A., Matysiak-Brynda E., Bystrzejewski M., Sutherland D.S., Stojek Z., Nowicka A.M. (2016). Conformational control of human transferrin covalently anchored to carbon-coated iron nanoparticles in presence of a magnetic field. Acta Biomater..

[48] Sahoo S.K., Labhasetwar V. (2005). Enhanced Antiproliferative Activity of Transferrin-Conjugated Paclitaxel-Loaded Nanoparticles Is Mediated via Sustained Intracellular Drug Retention. Mol. Pharm..

[49] Shao Z., Shao J., Tan B., Guan S., Liu Z., Zhao Z., He F., Zhao J. (2015). Targeted lung cancer therapy: Preparation and optimization of transferrin-decorated nanostructured lipid carriers as novel nanomedicine for co-delivery of anticancer drugs and DNA. Int. J. Nanomed..

[50] Lin X., Yan S.-Z., Qi S.-S., Xu Q., Han S.-S., Guo L.-Y., Zhao N., Chen S.-L., Yu S.-Q. (2017). Transferrin-modified nanoparticles for photodynamic therapy enhance the antitumor efficacy of hypocrellin a. Front. Pharmacol..

[51] Guo Y., Wang L., Lv P., Zhang P. (2015). Transferrin-conjugated doxorubicin-loaded lipid-coated nanoparticles for the targeting and therapy of lung cancer. Oncol. Lett..

[52] Liu K., Dai L., Li C., Liu J., Wang L., Lei J. (2016). Self-assembled targeted nanoparticles based on transferrin-modified eight-arm-polyethylene glycol-dihydroartemisinin conjugate. Sci. Rep..

